# Mechanisms and Applications of Manganese-Based Materials in Tumor Immunotherapy

**DOI:** 10.3390/molecules31101704

**Published:** 2026-05-18

**Authors:** Xiaoqi Kong, Changyue Zhang, Haodong Hu, Ye Chen, Wenjuan Gao, Ruijiao Chen

**Affiliations:** 1 School of Pharmacy, Shandong Medical and Pharmaceutical University, Yantai 264003, China; 2 College of Medical Imaging and Laboratory, Jining Medical University, Jining 272067, China; 3 School of Pharmacy, Shandong University of Traditional Chinese Medicine, Jinan 250355, China

**Keywords:** immunogenic cell death, manganese, nanomaterials, reactive oxygen species, tumor immunotherapy

## Abstract

Manganese-based nanomaterials have been novel multifunctional platforms in tumor immunotherapy because of their tunable multivalent states, biocompatibility, and multi-stimulus responsiveness. Current cancer treatments are insufficient and cause severe side effects; therefore, manganese-based nanomaterials are proposed in combination with immunotherapy to mitigate adverse effects. This review outlines the antitumor effects mediated by four key mechanisms: (1) activation of the cGAS-STING immune signaling pathway, (2) direct activation of immune cells, (3) induction of immunogenic cell death (ICD), and (4) modulation of the tumor microenvironment. These approaches are broadly categorized into two types: monotherapy and multimodal combination therapy. Monotherapy encompasses three specific modalities: (1) direct use as a Stimulator of Interferon Genes (STING) agonist, (2) vector-mediated targeted drug delivery, and (3) mediation of chemodynamic therapy to generate reactive oxygen species, thereby inducing ICD. Multimodal combination therapy involves synergistic integration with traditional or emerging treatment modalities, including chemotherapy, radiotherapy, photodynamic therapy, sonodynamic therapy, and low-level light therapy, as well as multimodal combination treatment methods. It significantly enhances the antitumor efficacy of traditional therapies through immunostimulation, thus achieving synergistic breakthroughs in treatment efficiency and survival rate. Collectively, the multifunctional integration of manganese-based materials is a novel strategy for developing “self-adjuvant” immunotherapeutic platforms and investigating the clinical translation potential.

## 1. Introduction

Currently, malignant tumors (cancer) are a major threat to human health [1,2]. Cancer is characterized by high morbidity and mortality, and its treatment methods have not been completely overcome, which makes many people “turn pale at the mention of cancer.” Many methods are available for tumor treatment. However, traditional methods, such as surgery, chemotherapy, and radiotherapy (RT), have many limitations, including injury to normal tissues, tolerance, and a high relapse rate [3]. Additionally, tumors are complex and heterogeneous, making it difficult for traditional single-drug therapies to completely eradicate them [4,5]. Immunotherapy has the potential to effectively kill metastatic tumor cells by activating the body’s own immune system, which could become the optimal strategy for treating tumor metastasis. The multimodal synergistic therapies involved in immunotherapy hold promise for eradicating tumors [6]. Immunotherapy can be applied to various cancer types, especially in patients that experienced poor efficacy with some traditional treatment methods [7]. It uses the body’s own immune system to attack cancer cells, which has a long-lasting effect and may produce significant therapeutic effects on some patients with advanced cancer. Antitumor immunotherapies include immune checkpoint blockade, tumor vaccines, CAR-T therapy, and programmed death receptor 1 (PD-1) immunotherapy [8,9,10]. However, low antigen presentation, immunogenicity, T-cell infiltration, and tumor immune tolerance may occur [11]. The use of nanomaterials with immunotherapy has been proposed to regulate the tumor microenvironment (TME), increase antigen presentation, enhance T cell expansion, and optimize tumor immunotherapy.

The application of manganese (Mn)-based materials includes various treatment methods, such as single application and multimodal synergistic therapy. As an essential trace element in the human body, Mn is involved in physiological processes, such as the host immune system, hematopoiesis, endocrine function, and oxidative stress regulation [12]. Mn-based nanomaterials with unique physicochemical properties, such as good biocompatibility, controllable size, special surface properties, and diverse potential for functional modification, are widely used in nanomedicine and have broad application prospects in tumor immunotherapy [13,14]. First, Mn-based nanoparticles (NPs) with good biocompatibility are nanocarriers for the delivery of immunomodulators. The nanomaterials protect immunosuppressive agents from degradation, increase cellular uptake, and enhance tumor immunotherapy. Second, Mn-based materials can be used as adjuvants to regulate the TME and enhance immunity. In immunotherapy, Mn directly activates cyclic GMP-AMP synthase (cGAS) and induces the non-canonical catalytic synthesis of 2′3′-cGAMP, activates stimulator of interferon genes (STING), and stimulates the immune system. The safety of treatment methods is becoming increasingly important with advancements in tumor immunotherapy research. Mn-based materials have therapeutic effects and are therapeutically safe. MnO_2_ is a multifunctional therapeutic agent for improving tumor treatment [15]. Therefore, exploring Mn-based nanomaterials has important scientific significance and clinical value and is expected to provide more effective treatment options for cancer patients and promote further developments in tumor immunotherapy.

## 2. Characteristics and Functional Advantages of Mn-Based Materials

### 2.1. Biological Characteristics of Mn

Mn, a transition metal, exists primarily in living organisms as Mn^2+^ and Mn^3+^; Mn^2+^ is more stable; however, while Mn^3+^ is a strong oxidizing agent [12,16]. It is also an essential trace element for mammals and plays a broad role in physiological processes, such as development, neural function, and antioxidant defense [17]. Its blood concentration is maintained at 4.8–8.3 μg/L, where it functions as a component of metalloproteins and an enzyme activator [18,19,20]. Its physiological distribution is organ-specific, with higher concentrations in bones and the liver and lower concentrations, such as <20 nmol/g, in brain tissue [21,22]. Mn is absorbed through multiple routes, including the veins, skin, gastrointestinal tract, and respiratory tract. After entering cells via passive diffusion or active transport, it primarily accumulates in the mitochondria and cell nucleus and is distributed via the bloodstream to tissues, such as bones, the liver, and the pancreas. Mn metabolism operates in dynamic equilibrium; excess amounts are conjugated by the liver and gallbladder, excreted into the intestines, and ultimately excreted from the body via feces within 10 days [23]. Even though manganese-based materials have low toxicity, to avoid metal deposition, water-soluble chelating agents (such as CaEDTA) can be combined to promote urinary excretion, and brain-targeting shuttle agents can be supplemented to enhance metal mobilization. Studies have confirmed that CaEDTA can effectively remove Mn, and the combination of PAS and CaEDTA can specifically target manganese deposition in the central nervous system and significantly improve the removal efficiency [24]. Mn can be used to enhance vaccine immunogenicity in tumor treatments. The Sq@TA/Mn@OVA vaccine enabled the co-delivery of Mn^2+^, Sq, and antigens. It is safe, enhances reactive oxygen species (ROS) generation and dendritic cell uptake, and improves antigen retention at the injection site [25]. Zhou et al. pioneered the development of a pH-sensitive Mn_3_(PO_4_)_2_·3H_2_O NPs as a novel bivalent COVID-19 protein vaccine adjuvant. Through its unique pH-responsive properties and long-term stability, it significantly enhances the broad-spectrum immune response of vaccines, outperforming traditional adjuvants [26]. As a component and activator of various enzymes, Mn participates in and regulates many biochemical reactions within the body, and plays a crucial role in modulating immune responses that collectively contribute to anticancer therapy.

### 2.2. Physical Properties of Mn

The physical properties of Mn are primarily manifested in its paramagnetism, density, particle size controllability, enzyme activity, and signal transduction characteristics, driven by its multivalent nature. Owing to their unique physicochemical properties and biocompatibility, Mn-based nanomaterials have significant advantages in drug delivery systems and are commonly used as highly efficient carriers of anticancer drugs.

Due to its paramagnetic properties, Mn^2+^ can significantly shorten the longitudinal relaxation time (T_1_), thereby enhancing signal intensity and positive contrast in T_1_-weighted magnetic resonance imaging (MRI) [27,28]. This property makes Mn^2+^ an effective MRI contrast agent, particularly suitable for tumor-specific imaging and detection [29,30,31,32]. Furthermore, upon binding to proteins, the relaxation rate of Mn^2+^ further increases, amplifying the MRI contrast signal [33]. Hu et al. introduced a DNA-Mn hydrogel that specifically targets thyroglobulin. This nanogel was loaded with paramagnetic Mn^2+^ to facilitate MRI [34]. The MnO_2_/CS@5-ALA-MTX NP system has demonstrated significant potential for the accurate diagnosis of cancers with overexpressed folate receptors, such as glioblastoma [35]. Furthermore, novel multifunctional nanoplatforms, such as CSF1R-IN-3@Mn@MPDA-antiTREM2, reprogram tumor-associated macrophages (TAMs) and have MRI-guided immune monitoring capabilities [36]. Mn-based contrast agents significantly enhance MRI contrast through tumor-specific accumulation, enabling precise localization of tumor boundaries and the extent of infiltration, offering significant advantages over traditional imaging methods [37]. Some Mn-based nanomaterials (such as MnO_2_) have photothermal conversion ability and ultrasonic response ability, which can be used to assist sonodynamic therapy (SDT) and photothermal therapy (PTT). This characteristic makes Mn-based materials potentially valuable for tumor ablation and local hyperthermia.

Compared with gadolinium-based contrast agents (GBCAs), manganese-based MRI systems have significant advantages in terms of biosafety, signal enhancement, and metabolic pathways. However, they still face challenges in stability and ligand design. GBCAs pose a risk of nephrogenic systemic fibrosis (NSF) and the problem of gadolinium deposition in the brain, especially for patients with impaired renal function [38,39]. As an essential trace element, manganese can be rapidly cleared by the liver without the risk of NSF [40]. A research team introduced a pentadentate ligand composed of two picolinate units and a rigid 1,2,3,4-tetrahydroquinazoline unit, as well as its corresponding diaqua manganese (II) complex. This complex exhibits high thermodynamic stability and kinetic inertness, comparable to the clinically used gadolinium (III)-based contrast agent Magnevist [41]. In vivo experiments on healthy mice showed that after intravenous injection of 0.08 mmol/kg of this compound, the magnetic resonance imaging (MRI) signal intensity of liver blood vessels was significantly enhanced, and the visualization of the gallbladder, kidneys, and liver was also improved. Although the experimental results of the manganese-based MRI system are relatively promising at present, there are still limitations. For example, the coordination stability is poor and needs to be improved with the help of chelating agents or nanocarriers [42,43]. Moreover, the preferential uptake by hepatocytes may affect the imaging quality of other organs. Currently, GBCAs have been maturely applied, while manganese-based contrast agents are still in the early stage of clinical research, and their long-term safety still needs to be verified. In the future, further research and development are needed to fully exploit the potential of manganese-based materials.

### 2.3. Chemical Properties of Mn

The core chemical properties of Mn are its ability to switch between oxidation states and its diverse catalytic activity, which make it a key molecule for regulating the TME, enhancing immune responses, and directly killing tumor cells. Mn exists in living organisms in various oxidation states, including Mn^2+^, Mn^3+^, and Mn^4+^, among which Mn^2+^ is the most stable and biologically active. In the TME, Mn^2+^ can undergo a Fenton-like reaction with H_2_O_2_ overexpressed within tumor cells, generating highly reactive hydroxyl radicals that directly damage tumor cell DNA, proteins, and lipid membranes, thereby inducing apoptosis or necrosis [44]. In the acidic environment within the tumor, Mn(IV) MnO_2_ can undergo a reduction reaction to form Mn(II)-ions (Mn^2+^), which catalyze the decomposition of H_2_O_2_ to produce O_2_, thereby alleviating hypoxia in the TME and enhancing sensitivity to RT and chemotherapy. This catalytic action enhances oxidative stress and depletes glutathione (GSH) within tumor cells, reducing their ability to scavenge ·OH radicals and amplifying oxidative stress-induced damage [45]. In summary, through its chemical properties, including valence state conversion and catalytic activity, Mn plays multiple roles in tumor therapy, such as improving the tumor microenvironment, enhancing oxidative stress, disrupting energy metabolism, and improving the efficacy of chemotherapy, thereby providing strong support for the development of novel tumor treatment strategies.

### 2.4. Functional Merits and Limitations: Benchmarking Manganese-Based Versus Alternative Anticancer Materials

Manganese-based materials (such as MnO_2_ NPs) activate the cGAS-STING pathway by releasing Mn^2+^ in tumor treatment, significantly enhancing the innate immune response. Mn^2+^ can lower the detection threshold of tumor-derived DNA, promote the maturation of antigen-presenting cells, and activate CD8^+^ T cells and NK cells, thereby inhibiting tumor metastasis and creating an anti-tumor microenvironment. In various mouse models, manganese-based materials have shown the effect of blocking lung metastasis. In contrast, copper-based materials rely on photothermal conversion or Fenton-like reactions to generate ROS [46], but the premature release of copper ions (Cu^2+^) may lead to high cytotoxicity, and technologies such as liposome encapsulation are required to control the release [47,48]. The degradation products of magnesium-based materials inhibit tumor growth by disrupting the energy metabolism of cancer cells and inducing oxidative stress, but they are mainly used in bone tumors and breast cancer, and their mechanism focuses on local environment regulation rather than systemic immune activation [49]. Although the degradation products of magnesium-based materials have low toxicity, the rapid degradation of magnesium alloys may lead to an increase in local pH and trigger an inflammatory response [50,51]. Iron-based materials generate reactive oxygen species through the Fenton reaction, but they rely on H_2_O_2_ in the tumor microenvironment, and iron overload can exacerbate oxidative stress and damage normal cells [52,53,54].

In terms of safety, as an essential trace element in the human body, Mn^2+^ can be rapidly excreted through the kidneys, and its biocompatibility is better than that of copper-based materials, with high safety in short-term treatment. However, excessive manganese accumulation may lead to neurotoxicity (such as Parkinson’s disease-like symptoms), so it is necessary to strictly control the dosage and release kinetics to avoid long-term neurotoxicity. In addition, surface modification with polyethylene glycol (PEG) or glucose transporter ligands can regulate the particle size to be greater than 20 nm, reducing the risk of Mn^2+^ entering the brain tissue. The premature release of Cu^2+^ in copper-based materials may lead to mitochondrial oxidative stress, and technologies such as liposome encapsulation are required to control the release [55]. The rapid degradation of magnesium-based materials may lead to an increase in local pH and trigger an inflammatory response, and alloying is required to delay the degradation [56,57]. The Fenton reaction of iron-based materials relies on H_2_O_2_ in the tumor microenvironment, and iron overload can exacerbate oxidative stress and damage normal cells.

In terms of cost and scalable production, manganese is abundant in reserves and low in price. For example, the cost of MnO_2_ precursors is much lower than that of gold or platinum-based materials. There are various synthesis methods for manganese-based materials, including hydrothermal methods, solvothermal methods, chemical precipitation methods, etc., which are more conducive to large-scale production. Although copper salts are inexpensive, the nanonization process requires high-temperature and high-pressure conditions, resulting in high energy consumption [58]; the processing of magnesium alloys requires strict control of the corrosion rate, and surface modification increases their cost; iron oxides are low in cost, but functional modification is required, which may increase the cost [59]. Currently, the main problems faced by manganese-based materials include the improvement of catalytic efficiency and the optimization of dosage and release control.

## 3. Mechanism of Antitumor Immunotherapy of Mn-Based Nanomaterials

### 3.1. Activation of STING Pathway

Mn^2+^ is the second activator of the cGAS-STING pathway besides double-stranded DNA (dsDNA) [60]. Mn plays three key roles in the cGAS-STING pathway: first, it effectively activates cGAS; second, it increases the sensitivity of the cGAS-STING signaling pathway to dsDNA; and third, it enhances the affinity between cGAMP and STING on the endoplasmic reticulum surface.

Mn^2+^ can effectively activate cGAS and, by binding to the STING protein, trigger the downstream TBK1-IRF3 signaling axis, which can stimulate host cells to produce type I interferons (IFN) without any infection [61]. Type I IFNs have immunomodulatory functions, can induce antineoplastic responses through CD8^+^ T and natural killer (NK) cells, and are crucial for the activation, proliferation, and effector functions of tumor-specific T cells [62,63]. Mn protects against DNA viruses by increasing the sensitivity of the cGAS-STING pathway to dsDNA [64]. When a virus invades the human body, Mn^2+^ ions are released from organelles, accumulate in the cytoplasm, and bind to cGAS, thereby generating an antiviral immune response [11]. Notably, Mn^2+^ can enhance the ability of cGAS to catalyze the cGAMP generation at low dsDNA levels, enhance the affinity of cGAMP and STING on the endoplasmic reticulum surface, induce IRF3 phosphorylation, activate the NF-κB pathway, and promote the production of IFN-1 [65,66]. Additionally, Mn-based materials can promote the secretion of IFN-γ (IFN-γ) [67]. IFN-γ plays an important role in antiviral, anti-tumor and immunization regulation. It activates macrophages, enhances their phagocytic and bactericidal abilities, and regulates the functions of other immune cells. Mn-based materials also inhibit immunosuppressive cytokine production. Additionally, they create a microenvironment conducive to immune cell function by inhibiting immunosuppressive molecules. The effect of Mn^2+^ on the immune system through this pathway is directly mediated by Mn^2+^ itself.

### 3.2. Direct Activation of Immune Cells

Mn^2+^ significantly enhances the activation and function of immune cells through both direct and indirect mechanisms, thereby promoting antitumor immune responses. First, Mn^2+^ promotes the maturation of dendritic cells (DCs) and macrophages. By activating the NF-κB signaling pathway, it enhances the ability of DCs to express antigen-presenting molecules, enabling them to more effectively uptake, process, and present tumor-specific antigens to T lymphocytes, thereby initiating an immune response [68,69]. Chen et al. synthesized the Mn ferrite nanohybrid MnFe_5_O_8_@(M1M-DOX), which activates the cGAS-STING and NF-κB pathways in DCs and TAMs, thereby stimulating innate and adaptive immune responses and reversing the tumor-suppressive microenvironment [70]. For example, the SV@BMs complex developed by Zhang et al. releases Mn^2+^ in response to GSH, triggering a Fenton-like reaction in macrophages. In synergy with the TLR7 agonist 1V209, this activates the NF-κB pathway, induces tumor cell apoptosis, and significantly increases the proportion of pro-inflammatory macrophages and T-cell infiltration [71]. Furthermore, Mn^2+^ can enhance the differentiation and activation of cytotoxic T lymphocytes (CTLs) as well as the activation of NK cells, thereby increasing their cytotoxic activity by regulating the balance between activation and inhibitory receptors on the surface of NK cells [68]. Mn^2+^ also increases the number of memory CD8^+^ T cells. By activating CD8^+^ T and NK cells, it eliminates tumors that are both sensitive and resistant to CD8^+^ T cells, promotes antitumor immune responses, and enables the rapid response and efficient elimination of tumor cells upon subsequent tumor recurrence. Mn-based materials also enhance the ability of T cells to recognize and respond to antigens by directly regulating the T cell receptor (TCR) signaling pathway. Simultaneously, they create more favorable conditions for T-cell activation by promoting maturation and antigen presentation in DCs. This enhances T cell recognition of tumor antigens within the TME and activates CTLs to perform their tumor-killing function. This multi-level mechanism of direct immune cell activation makes Mn-based materials ideal modulators for enhancing antitumor immune responses.

### 3.3. Induction of Immunogenic Cell Death (ICD)

Immunogenic cell death (ICD) is a form of programmed cell death that releases damage-associated molecular patterns (DAMPs) by dying cells. These molecules promote the activation of the congenital and adaptive immune system, thereby breaking tumor immune tolerance and inducing systemic antitumor immune responses. Mn-based nanomaterials can generate ROS through Fenton-like reactions, trigger endoplasmic reticulum stress and DAMPs release, activate DCs, and promote antigen-presenting ICD. Zhao et al. innovatively designed a Mn-enhanced catalytic immunotherapy that could be activated by the TME. This therapy uses tumor cell membrane (CM)-encapsulated multienzyme-mimetic Mn oxide nanoenzymes (abbreviated as CM@Mn), which exhibit unique peroxidase and coproporphyrinogen activity in the acidic TME, enabling in situ generation of hydroxyl radicals (•OH) and superoxide radicals (•O^2−^) to achieve dual tumor killing and induce ICD [72]. ICD is the result of tumor cell death and immunization therapeutic strategy, which can transform a “cold tumor “ (immunosuppression) into a “hot tumor “ (immune system activation).

### 3.4. Modulation of TME

Tumors reshape their mesenchymal environment to support their growth, which establishes the TME [73]. Mn alters the TME and promotes the infiltration of immune cells into tumor tissues. By regulating the function of tumor vascular endothelial cells or the secretion of chemokines, it attracts immune cells (such as T cells, NK cells, and macrophages) into tumor tissues, which increases the number of immune cells in tumor tissues and enhances the contact between immune and tumor cells, thereby improving the killing effect of immune cells on tumor cells. NK cells are important immune cells that rapidly lyse certain tumor cells and play a key role in immune surveillance. In a neoplastic microenvironment, hypoxia affects the ability of natural killer cells to undergo metastasis. Taking advantage of this phenomenon, Murphy et al. embedded MnO_2_ NP in poly (lactic-co-glycolic acid) to catalyze the degradation of H_2_O_2_ in tumors and generate O_2_, thereby promoting the adoptive transfer of NK cells and influencing the phenotype of tumor-associated macrophages [74]. Mn^2+^ released by Mn-based materials can also activate pattern recognition receptors in macrophages, trigger the NF-κB signaling pathway, and induce the expression of M1-type markers (iNOS and TNF-α). iNOS catalyzes the generation of nitric oxide (NO), which can directly kill tumor cells [75], and IL-12 can promote the activation of T and NK cells. The increased concentration of GSH in the TME promotes the decomposition of MnO_2_ NP and the release of Mn^2+^, thereby increasing the expression of iNOS in the TME. A dual-cascade nano-booster based on MnO_2_ NP was prepared for deep tumor immunization therapy [76].

During the development of TAMs are crucial for the growth of various experimental tumors in vivo and they are usually polarized into an ME-type-dominated phenotype. However, M2-type macrophages have immunosuppressive and pro-tumor functions, promoting blood vessel formation, tumor invasion, and metastasis [77,78]. Compared with M1-type macrophages, M2-type macrophages exhibit significantly higher expression of tumor-related cytokines (such as TNF-α and IL-10), accompanied by the inhibition of the secretion of the antitumor effector factor, IL-12, and upregulation of the hypoxia-inducible factor, HIF-1α. This phenotypic difference shows a clear pro-tumor tendency in the tumor microenvironment [79,80,81]. Mn-based nanomaterials can reprogram M2 cells into M1 cells with pro-inflammatory and antitumor functions by regulating key signaling pathways [82,83]. Additionally, Mn^2+^ can promote STAT1 phosphorylation, enhance IFN-γ signal response, and further consolidate M1 polarization, thereby reversing the immunosuppressive microenvironment.

**Figure 1:**
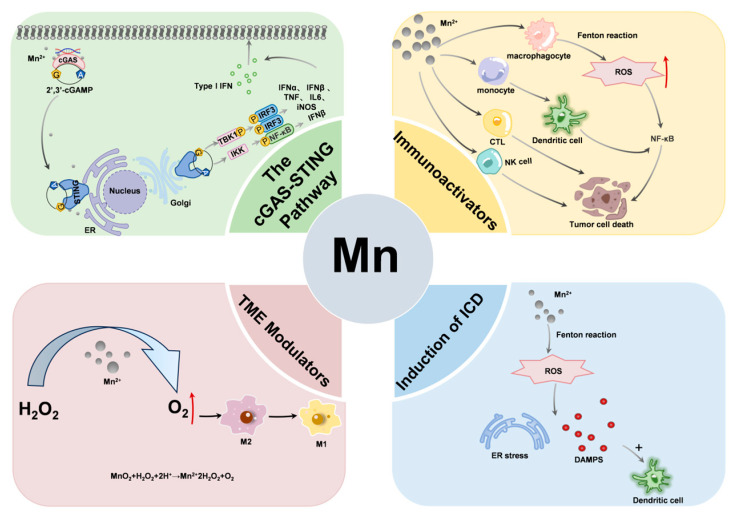
Immunotherapeutic Mechanism of Manganese (Mn)-Based Nanomaterials Against Tumors.

In general, manganese-based nanomaterials synergistically enhance anti-tumor immunity through ICD induction, STING activation, and TME regulation (Figure 1). Manganese-based nanomaterials release Mn^2+^ in an acid-responsive manner. Mn^2+^ competitively replaces Zn^2+^ at the Zn2 site (Cys416/Cys418) in the catalytic core of cGAS through its octahedral coordination advantage, driving cGAS-DNA phase separation to enhance cGAMP synthesis and promoting the palmitoylation of the STING dimer (Cys91), thereby activating the TBK1-IRF3 axis to produce IFN-β. At the same time, manganese-based nanomaterials (such as MnO_2_) catalyze H_2_O_2_ to generate O_2_, alleviating the hypoxic state in the TME, degrading HIF-1α, and inhibiting the functions of myeloid-derived suppressor cells (MDSC) and regulatory T cells. In addition, Mn^2+^ enhances the level of ROS by consuming GSH, further promoting ICD and STING signal amplification. The synergistic effect of these mechanisms not only enhances the maturation and antigen presentation ability of DCs, but also promotes the activation of CD8^+^ T cells and the formation of memory T cells, thus achieving a long-lasting anti-tumor immune response.

## 4. Applications of Mn-Based Nanomaterials in Tumor Immunotherapy

### 4.1. Single Treatment

#### 4.1.1. Mn-Based Nanocarrier

Immunotherapeutic agents, such as antibodies and genes, are biologically active and easily degraded by enzymes when used alone. A common approach to address this issue is the use of nanostructured biomaterials as drug-delivery carriers [84]. Compared to other materials, Mn-based nanomaterials have a larger specific surface area, exceptional performance, and diverse functionalities, providing substantial potential for development, particularly in tumor diagnosis and treatment. Currently, various Mn-based nanomaterials (such as MnO_2_, Mn_3_O_4_, and metal–organic frameworks) have entered the preclinical research stage [85]. Mn-based nanocarriers enable precise drug delivery based on their properties. The MnO_2_@CeO_2_-Vin-Ser nanocatalyst is prepared by successfully incorporating cerium oxide (CeO_2_), vincristine (Vin), and sericin (Ser) into MnO_2_. The pH-responsive drug delivery enhanced by serine and the synergistic catalysis of CeO_2_-MnO_2_ significantly enhance the cytotoxic effect on cancer cells. Moreover, ROS measurement shows that MnO_2_@CeO_2_ can induce oxidative stress, while sericin can alleviate the excessive production of ROS [86]. Wu et al. synthesized Mn-ZIF-8 nanoparticles by combining zinc nitrate and 2-methylimidazole, loaded the drug molecule RRX-001, and performed surface modification with PEG-HA to obtain Mn-ZIF-8@RRX-001@PEGHA (MRPH) nanoparticles. The MRPH nanoplatform synergistically activates the cGAS-STING pathway through pH-responsive release of Mn^2+^ and NO. Mn^2+^ enhances the mtDNA detection, and NO induces mitochondrial damage. Concurrently, targeted delivery promotes ICD, DC maturation, and CD8^+^ T cell infiltration, and inhibits CD47-mediated immune evasion [87]. Chu et al. investigated a hybrid platform (MS@Yeast) constructed by “armoring” the surface of live yeast with Mn silicate compounds, in which Mn^2+^ reacts with CO_2_ and induces intense oxidative stress in tumor cells, thereby ablating primary tumors [88]. Wen et al. coated a chitosan-opalin core loaded with metformin with MnO_2_ to create CS-metformin@MnO_2_, which was used to inhibit the PD-1/PD-L1 signaling pathway and enhance tumor immunotherapy [89]. The primary function of Mn-based nanocarriers is to prevent drug release and degradation before reaching the tumor site, thereby improving the bioavailability of the immunotherapeutic agent. Wen et al. synthesized H_2_O_2_-responsive bio-mimetic NPs (MnO_2_-ICG@BSA) by loading indocyanine green (ICG) onto bovine serum albumin-MnO_2_ complexes [90], establishing promising candidates for PTT and photodynamic therapy (PDT) in the treatment of melanoma. Manganese-based nanomaterials are also used in combination with other materials to treat tumors. Liu et al. effectively combined molybdenum with Mn to develop polyethylene glycol-modified Mn molybdate NPs(MMP NDs) for cancer immunotherapy [91], demonstrating that this combination therapy can achieve synergistic therapeutic effects in vivo. As demonstrated by the above examples, Mn-based nanomaterials play a critical role in tumor immunotherapy. They are primarily used for the targeted delivery of drugs, significantly improving the bioavailability of immunotherapeutic agents and enhancing the efficacy of tumor treatments, making them well-suited for immunotherapy.

#### 4.1.2. Mn^2+^-Mediated Chemodynamic Therapy (CDT)

Chemodynamic therapy (CDT) is a novel tumor treatment technology that relies on the characteristics of weak acidity and excessive H_2_O_2_ in the TME. It uses nano catalytic systems to trigger in-situ Fenton or Fenton-like reactions in tumors, converting H_2_O_2_ with originally low activity in tumor cells into highly •OH, and ultimately inducing cell apoptosis and necrosis [92]. CDT has low toxicity and few side effects in normal tissues, strong selectivity, suitability for the treatment of tumors deep in tissues, and unique advantages in tumor treatment.

Mn, as an essential metal in the human biological system, can be applied to the composition of CDT preparations. The •OH released from Mn-based nanomaterials by Mn^2+^ has Fenton-like activity and produces high toxicity, killing tumor cells through endogenous H_2_O_2_ [93]. Hydroxyl radicals have strong oxidizing properties and can oxidize biological molecules, such as tumor cell membranes, protein molecules, and DNA, leading to oxidative stress in neoplastic cells and triggering apoptosis. Among the Mn-based catalysts that mediate CDT, Mn oxide (MnOX) is the most common. For example, Li et al. developed a highly efficient, biocompatible PTX/MnO_2_/GOX-Lip-HAs nanodelivery system, in which paclitaxel (PTX), MnO_2_ NPs, and glucose oxidase self-assemble into nanospheres. This system exerts traditional chemotherapeutic effects and generates H_2_O_2_ and oxygen via MnO_2_, thereby depleting endogenous GSH and promoting starvation therapy, all of which enhance the efficacy of CDT [94]. In this system, in addition to exerting the traditional chemotherapy effect, MnO_2_ NPs also have the ability to generate H_2_O_2_, to produce oxygen, and consume endogenous GSH through redox reactions, thereby reducing the scavenging of •OH. Mn^2+^ can also participate in Fenton-like reactions, substantially enhancing the effect of the CDT. Additionally, the O_2_ generated during the CDT process promotes starvation therapy and provides more H_2_O_2_ to enhance CDT. Li et al. found that when this drug delivery method was applied to tumor-bearing mice, the final tumor inhibition effect was greater than 90% [94], providing important theoretical support for the translational application of Mn-based CDT in combined tumor immunization therapy. MIL-53(Fe)@MnO_2_ is stabilized with polyethylene glycol (FMP), capable of consuming GSH and continuously generating •OH, which is used to enhance the CDT for cancer [95]. The Gen@mSiO_2_@MnO_2_-PEG nanocomposites synthesized by Fan et al. consist of a mesoporous silica core and a MnO_2_ shell, and are loaded with the chemotherapeutic drug genistein (Gen), with its surface modified by PEG. It releases gold-enamide upon tumor delivery to perform CDT and degrade the MnO_2_ shell; GSH uptake triggers a Fenton-like reaction to generate •OH, which synergistically enhances the efficacy of CDT [96].

**Figure 2:**
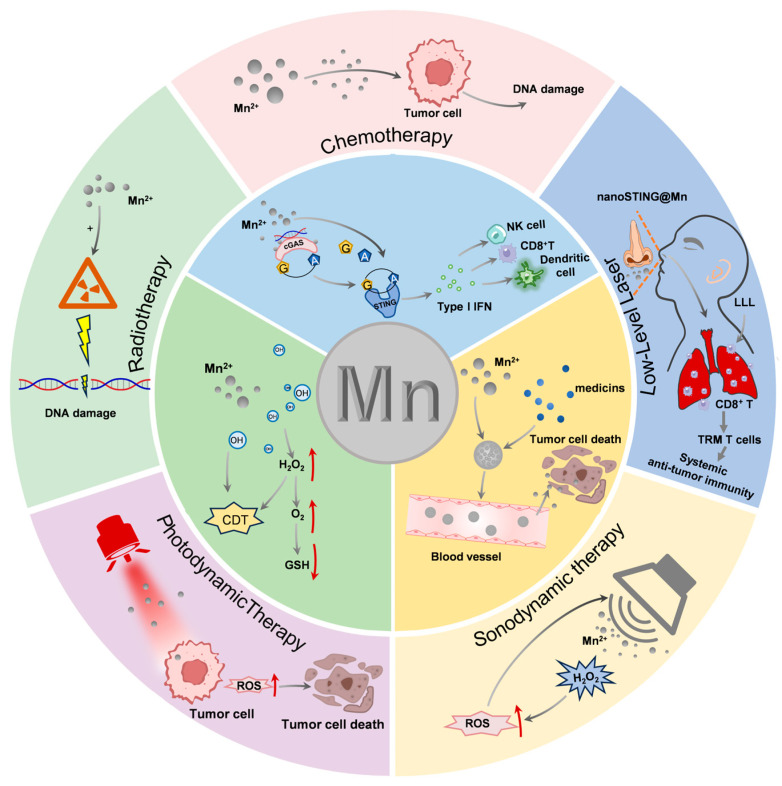
Manganese (Mn)-Based Nanomaterials in Monotherapy and Multimodal Synergistic Applications for Tumor Immunotherapy.

Therefore, Mn-based nanomaterials have broad application prospects in CDT and as multifunctional intelligent nanoplatforms, they have important value in achieving efficient tumor therapy (Figure 2).

#### 4.1.3. STING Pathway-Dependent Immune Activation

When a virus invades the body, it disrupts the mitochondrial membrane potential of host cells and causes organelle acidification, leading to the release of Mn^2+^ from Mn-containing organelles, such as mitochondria and the Golgi apparatus, into the cytoplasm and extracellular space. Elevated Mn^2+^ concentrations in the cytoplasm exert a dual activating effect on the cGAS-STING pathway. At the molecular level, Mn^2+^ acts on cGAS, enabling the body to defend against dsDNA in the cytoplasm by enhancing the sensitivity of cGAS to dsDNA detection and promoting its enzymatic activity. Additionally, it acts on STING, enhancing its affinity for various cyclic dinucleotides (CDNs) [11,97]. This dual activation triggers the unique catalytic synthesis of 2′3′-cGAMP, which promotes the substantia type 1 IFN production [65]. Type 1 IFNs enhance cellular immunity, stimulate the activation of DCs, and facilitate the cross-presentation of tumor antigens, thereby inducing antitumor T-cell immunity [98]. The role of Mn^2+^ in activating the cGAS-STING pathway and its immunotherapeutic effects has been established. For example, Liu et al. used MnO_2_ NPs to degrade adenosine (an immunosuppressive molecule) in the TME, and simultaneously released Mn^2+^ to activate the STING pathway, significantly enhancing T-cell activity [99]. As-MnZnSX NRs induce TBK1/IRF3 phosphorylation via Mn^2+^ to activate the cGAS-STING pathway, promoting DC maturation and cross-presentation of tumor antigens; H_2_S synergistically enhances prodrug conversion and immune activation, achieving dual stimulation of the cGAS-STING pathway and significantly improving the antitumor efficacy of As_2_O_3_ [100]. CMP Mn-based NPs (Mn^2+^/c-di-AMP) enhance STING pathway activation via Mn^2+^ and synergize with anti-CTLA-4 blockade, significantly increasing CD8^+^ T-cell infiltration and prolonging survival in a disseminated AML model, confirming the key synergistic role of Mn-based materials in combination immunotherapy [101]. Sulfur-based hybrid organosilicon materials were loaded with Mn^2+^ to enhance Mn^2+^ release during RT, thereby activating the STING pathway, enhancing the tumor immune response, and inhibiting tumor growth [102]. The TPP-MMONs nanoplatform induces mitochondrial DNA release through the targeted release of Mn^2+^, thereby dual-activating the cGAS-STING pathway and enhancing the efficacy of αPD-L1 immunotherapy, offering a new strategy for the treatment of triple-negative breast cancer [103]. Chen et al. reported a tumor cell membrane-coated biomimetic nanozyme platform (CMNP), which contains manganese dioxide nanoparticles (MnO_2_@BSA), nano-realgar (NR) and doxorubicin (DOX). Within tumors, MnO_2_ alleviates hypoxia and enhances enzyme activity by catalyzing the decomposition of H_2_O_2_ to generate O_2_, and the released Mn^2+^ recruits tumor-killing immune cells by activating the cGAS-STING pathway [104]. MnCREKA-aCTLA-4-SS (MCCS) is prepared by covalently assembling Mn^2+^, silk sericin (SS), pentapeptide CREKA, and aCTLA-4. By activating the cGAS-STING pathway, MCCS stimulates effector CD8^+^ and CD80^+^ T cells, increasing IFN-γ and granzyme secretion, thereby inducing tumor cell autophagy and apoptosis both in vitro and in vivo [105]. Mn^2+^ ions released by Mn-LDH-Ce_6_ activate the cGAS-STING pathway, promoting STING phosphorylation and IFN-mediated immunity, thereby effectively reversing immunosuppression and inhibiting tumor growth [106].

In summary, Mn^2+^, as a novel STING agonist, enhances antitumor and antiviral immune responses by activating the cGAS-STING pathway, thereby providing an important theoretical foundation for the development of novel immunotherapy strategies (Table 1).

### 4.2. Dual-Modal and Multi-Modal Collaborative Therapy

#### 4.2.1. Mn-Based Immunotherapy Combined with Chemotherapy

**Figure 3:**
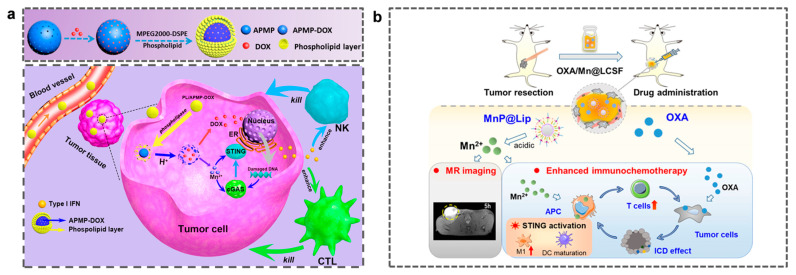
(**a**) Schematic illustration of the synthesis of PL/APMP-DOX and the multi-pathway mechanisms of synergy in tumor treatment achieved by combining chemotherapy with manganese-based immunotherapy. Reprinted (adapted) with permission from [107]. Copyright 2020, American Chemical Society (**b**) An injectable gel system co-loaded with MnP@Lip nanoparticles and oxaliplatin (OXA) can enhance immunochemotherapy and treatment pathways. The upward arrow in the figure indicates the increase of M1 and T cells. Reprinted (adapted) with permission from [108]. Copyright 2021, copyright Li J, Li S, Li Y, et al.

Chemotherapeutic drugs play a crucial role in cancer treatment. However, drug resistance limits their therapeutic efficacy. Mn-based nanomaterials activate the immune system and can effectively integrate chemotherapy with immunotherapy, thus offering a promising combination therapy strategy to overcome drug resistance. The combination of Mn-based nanomaterials and chemotherapy leverages the cytotoxic effects of chemotherapeutic agents to achieve a stronger direct killing effect. Hou et al. encapsulated doxorubicin (DOX) within amorphous porous Mn phosphate NPs and coated the outermost layer with phospholipids to form PL/APMP-DOX NPs. In an acidic TME, the exposed NPs degrade and rapidly release Mn^2+^ and DOX. The cytotoxic action of DOX causes DNA damage in tumors and activates the cGAS-STING pathway. Mn^2+^ further amplifies this activation, inducing type 1 IFN production, promoting DC maturation, and enhancing cytotoxic effects mediated by CD8^+^ T cells or NK cells (Figure 3a) [107].

Platinum-based compounds can also be combined with Mn-based nanomaterials. For example, Li et al. co-administered MR nanoprobes (MnP@LiP) with platinum-based chemotherapeutic agents, thereby inhibiting the growth of 4T1 and MC38 mouse tumor cells (Figure 3b) [108]. C-NAG-R8-PTXL/MnO_2_-lip enables MRI-guided synergistic chemotherapy (CDT/CT). The generated oxygen can also relieve the hypoxic environment within the tumor, thereby reducing the efflux of chemotherapeutic drugs [109]. Although the synergistic effect of Mn-based materials and chemotherapy has shown initial advantages by improving drug release efficiency and treatment persistence, with the increasing complexity of tumor heterogeneity and immunosuppressive microenvironment, the single synergistic mode has difficulty breaking through the efficacy bottleneck, prompting the research to shift towards multimodal synergistic strategies integrating treatments, such as RT and PDT.

#### 4.2.2. Mn-Based Immunotherapy Combined with RT

**Figure 4:**
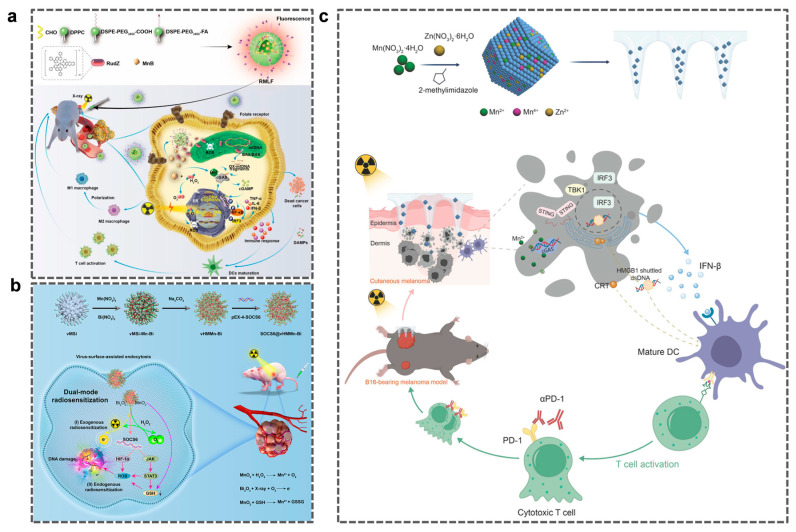
(**a**) Synthesis process of RMLF and its application in radioimmunotherapy of breast cancer. Reprinted (adapted) with permission from [110]. Copyright 2024, copyright Peng J, Quan D L, Yang G, et al. (**b**) Vi-rus-inspired SOCS6@vHMMn-Bi nanoparticles have dual-mode sensitization for enhancing radiotherapy of esophageal cancer. Reprinted (adapted) with permission from [111]. Copyright 2025, Wiley-VCH GmbH. (**c**) Schematic diagram of the preparation process of Mn-ZIF-8 and the mechanism pathway of mediating radiosensitization and stimulating the STING pathway to improve radioimmunotherapy. Reprinted (adapted) with permission from [112] Copyright. 2025, copyright Hu W, Hong X, Zhang X, et al.

RT is the optimal method for local tumor control after surgical resection and one of the primary clinical treatments for various malignant tumors. The primary mechanism involves the induction of ICD in tumor cells to elicit a systemic immune response. However, monotherapy with RT struggles to suppress distant metastasis owing to insufficient DAMP release and a hypoxic microenvironment. Mn-based immunotherapy overcomes this limitation through synergistic mechanisms. The RMLF nanoplatform integrates Ru(II) complexes and BSA-MnO_2_ NPs to achieve dual enhancement of radiation-induced cell accumulation, ROS generation, and cGAS-STING pathway activation (Figure 4a). This effectively promotes CTL infiltration and the polarization of macrophages from M2 to M1, significantly enhancing immune memory responses and overcoming the issue of insufficient DAMP release during conventional RT (4 Gy) [110]. Some researchers have developed a SOCS6@vHMMn—Bi nanoparticle, which is composed of virus—inspired hollow mesoporous manganese—bismuth bimetallic oxide nanoparticles (vHMMn-Bi) and a radiosensitizing plasmid (suppressor of cytokine signaling 6, SOCS6). It can significantly improve the efficacy of radiotherapy for esophageal cancer through a dual—mode radiosensitization mechanism. Among them, MnO_2_ catalyzes oxygen production, thus significantly improving the radiotherapy effect without systemic toxicity (Figure 4b) [111]. Mn-ZIF-8, a transdermal drug delivery system, enhances radiosensitivity in cutaneous melanoma when combined with RT and strengthens ICD by enhancing STING pathway activation (Figure 4c) [112]. Moreover, under the condition of pH 5.5, Mn^2+^ is almost completely released within 3 h, achieving responsive activation in the acidic tumor microenvironment. While at pH 7.4, the cumulative release rate is only 23% within 24 h, reducing leakage to normal tissues and avoiding adverse side effects. Liu et al. proposed that the αPDL1@MnO_2_ nanoplatform promotes DC maturation by releasing Mn^2+^ to activate the cGAS-STING pathway and, in combination with αPDL1, enhances CTL infiltration. This significantly improves the cytotoxic effects of RT on tumor cells, overcomes hypoxia-induced radiation resistance, and reprograms the immunosuppressive TME, thereby effectively inducing ICD and generating distant effects that inhibit tumor metastasis. [113]. Liu et al. reported a bio-mineralized Mn oxide NP (Bio-MnO_2_NPs) capable of converting endogenous H_2_O_2_ into O_2_ and catalyzing ROS production, thereby increasing the radiosensitivity of non-small-cell lung cancer cells. Concurrently, the release of Mn^2+^ activates the STING pathway and the combined therapy significantly inhibits tumor growth in lung cancer-bearing mice [114]. Furthermore, unlike combinations with chemotherapeutic agents, combining Mn^2+^ and RT may require special attention. Wang et al. found that the intratumoral injection of Mn^2+^ immediately after RT did not enhance RT, whereas intratumoral injection 24 h after RT did [115]. This is because Mn^2+^ injected directly is metabolized out of the tumor within minutes, whereas RT-induced DNA damage requires up to 24 h of cellular mitosis to accumulate in the cytoplasm and exert a synergistic effect with Mn^2+^. Therefore, the release time of NPs should also be considered during the design to coordinate with RT and achieve optimal therapeutic outcomes.

In summary, Mn-based immunotherapy significantly enhances the efficacy of RT through three mechanisms: oxygen sensitization, hypersensitization of the STING pathway, and remodeling of the immune microenvironment. By optimizing the timing of drug administration to achieve synergistic effects, it provides a new strategy for systemic anti-metastatic treatment of tumors.

#### 4.2.3. Mn-Based Immunotherapy and PDT

PDT is a non-invasive treatment method. When the photosensitizer is in an oxygenated state, light radiation can be precisely applied to the target tumor, generating toxic ROS, which chemically react with various biomolecules in the surrounding area, causing damage to subcellular structures, such as mitochondria, cell membranes, and lysosomes, and ultimately effectively induces cell apoptosis or necrosis [116,117,118]. During necrosis, tumor cells produce large amounts of antigens, thereby stimulating the body’s immune response. However, the limitations of conventional PDT are insufficient efficacy against metastatic and deep-seated tumors, and the fact that most photosensitizers are hydrophobic and non-selective, leading to non-specific damage [119,120,121]. To overcome these limitations, Mn-based nanomaterials are conjugated with traditional photosensitizers. By leveraging their hydrophilic properties and drug delivery capabilities, they achieve highly efficient targeted accumulation through the enhanced permeability and retention effect, significantly reducing damage to the surrounding healthy tissues [122,123,124,125,126].

**Figure 5:**
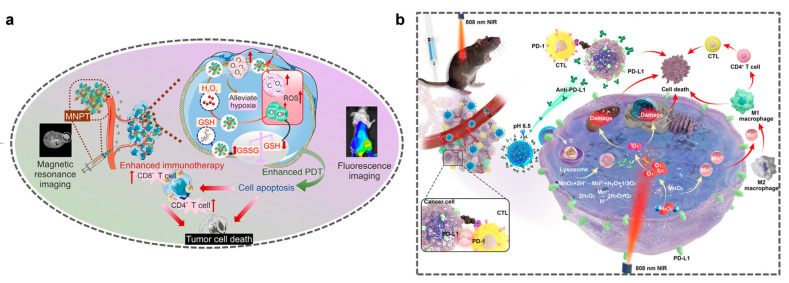
(**a**) Schematic diagram of MNPT enhancing tumor treatment through manganese-based immunotherapy and photodynamic therapy (PDT). Reprinted (adapted) with permission from [127]. Copyright 2024, copyright Bai C, Liu J, Bai L, et al. (**b**) Schematic diagram of ICG@MnO_2_@Exo-anti-PD-L1 for immune-remodeling photodynamic therapy in the treatment of non-small-cell lung cancer (NSCLC). Reprinted (adapted) with permission from [128]. Copyright 2024, copyright Guo J, Zhao W, Xiao X, et al.

For example, the magnetic nanoplatform (MNPT), comprising the photosensitizer, Ce_6_, and Fe_3_O_4_/MnO_2_ composite nanoenzymes, generates ROS via PDT to induce cell apoptosis and trigger an immune response (Figure 5a) [127]. MnO-N/C NPs activate their enzymatic activity via an 808 nm near-infrared laser, enabling photothermal-enhanced catalytic-photothermal synergistic therapy under MRI guidance, thereby providing a new strategy for low-toxicity, high-efficiency tumor nanotherapy (Figure 5b) [128]. ICG@MnO_2_@Exo-anti-PD-L1 NPs effectively regulate the TME to combat non-small-cell lung cancer through the synergistic effect of photodynamic therapy and immunotherapy. These NPs are prepared by loading indocyanine green (ICG) into hollow manganese dioxide nanoparticles (ICG@MnO_2_), and then encapsulating them in exosomes derived from dendritic cells (DCs) treated with azidoethylcholine (AECho) [129]. The PDA@Mo_2_C-MnO_2_-Au/Apt-M cascade nanoreactor triggers MnO_2_-mediated Fenton-like reactions (•OH generation) in an acidic TME, which synergizes with near-infrared (NIR)-II photothermal effects to achieve targeted PTT/CDT combination therapy [130]. The FM@VP nanoplatform developed by Chung et al. is based on the co-assembly of nanocomposites formed by the polysaccharide fucoidan, the bioreducible polyamidoamine (PAMAM) dendrimer, the photosensitizer verteporfin (VP), and MnO_2_ nanoparticles into multifunctional nanoparticle clusters. By using MnO_2_ nanoparticles to relieve tumor hypoxia and synergistically inhibiting the Yes-associated protein (YAP) signaling pathway through VP—mediated photodynamic therapy, it significantly enhances the anti—tumor immune response and improves the efficacy of photodynamic therapy [131]. Gao et al. considered the problems such as the excessive release of reactive oxygen species (ROS) and the resulting damage to non-target tissues, and developed an innovative hybrid nanorobot (EcN+UCNPs@MSiO_2_-MnO_2_-ZnPc), which integrates engineered Escherichia coli Nissle 1917 (EcN) with orthogonally emitting upconversion nanoparticles (C@3S) [132]. The manganese-loaded upconversion nanoparticles (UCNPs@Mn) are triggered by 808 nm near-infrared light to generate ROS, which increases the ROS level in the illuminated area and leads to a significant decrease in cell viability. After the light is turned off, the ROS generation rate decreases significantly, thus achieving spatially precise killing.

By precisely regulating the spatiotemporal synergy between PDT and immunotherapy, these strategies address the issue of insufficient photosensitizer targeting and overcome the therapeutic bottlenecks of PDT for metastatic and deep-seated tumors, thereby providing a new paradigm for combined cancer therapy.

#### 4.2.4. Mn-Based Immunotherapy and SDT

**Figure 6:**
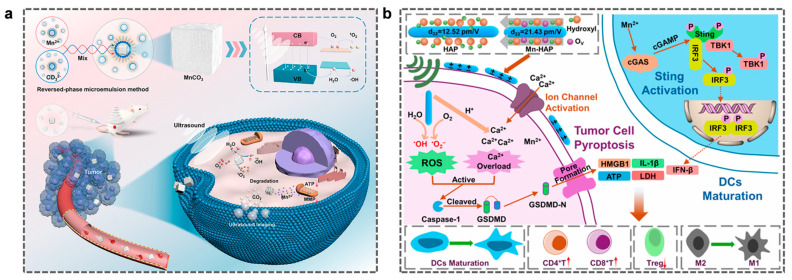
(**a**) Schematic diagram of the synthesis of manganese carbonate nanoparticles and anti—tumor treatment through enhanced SDT. The upward arrow in the figure indicates an increase in CD4+ T and CD8+ T cells, while the downward arrow indicates a decrease in Tregs. Reprinted (adapted) with permission from [134]. Copyright 2021, copyright Zhang H, Pan X, Wu Q, et al. (**b**) Schematic diagram of the anti-tumor process and immune response of manganese-hydroxyapatite. Reprinted (adapted) with permission from [135]. Copyright 2025, American Chemical Society.

In cancer therapy, SDT is an emerging non-invasive treatment modality that induces tumor cell apoptosis using ultrasound to activate photosensitizers and generate cytotoxic ROS [133]. However, a hypoxic TME and excessive GSH levels lead to insufficient ROS production, thereby limiting the antitumor efficacy of SDT. Mn^2+^ can interact with H_2_O_2_ in the TME to generate ROS via the Fenton reaction, thereby enhancing the efficacy of SDT. This effectively addresses these limiting factors and establishes avenues for the clinical application of SDT. Recently, Zhang et al. developed highly efficient Mn carbonate NPs (MnCO_3_ NPs) as photosensitizers using a reverse microemulsion method to enhance SDT (Figure 6a) [134]. Under ultrasonic conditions, MnCO_3_ NPs can generate •OH and singlet oxygen. Furthermore, due to their pH responsiveness, MnCO_3_ NPs can degrade to release CO_2_ and Mn^2+^ in an acidic microenvironment. The resulting CO_2_ bubbles enhance the cavitation effect under ultrasound, thereby mediating the irreversible necrosis of cancer cells and conferring ultrasonic imaging capabilities. In vivo experimental data show that MnCO_3_ NPs inherently possess a tumor inhibition rate of approximately 50%; following ultrasonic stimulation, the tumor suppression efficiency significantly increases up to 90%. The SDT synergistic anticancer strategy proposed in this study broadens the scope of photosensitizers and provides new insights and perspectives for the development of nanomedicine.

Mn-HAP is a photosensitizer and an immunomodulator that triggers the continuous modulation of ROS via ultrasound and enhances the sonodynamic antitumor effect by using Mn-doped oxygen vacancies (Figure 6b). In this process, Mn^2+^ activates the cGAS-STING pathway, thereby stimulating innate immunity and inducing antitumor immune responses [135].

These innovative studies confirm that Mn-based materials, through a triple synergistic mechanism involving catalytic oxygenation, immune activation, and physical enhancement, not only overcome the therapeutic bottlenecks of traditional SDT, but also provide a crucial theoretical foundation and technical support for the development of novel SDT-immunotherapy combination strategies. With continuous advancements in nanotechnology, this synergistic treatment model is expected to advance clinical cancer therapy.

#### 4.2.5. Mn-Based Immunotherapy and Low-Intensity Phototherapy

**Figure 7:**
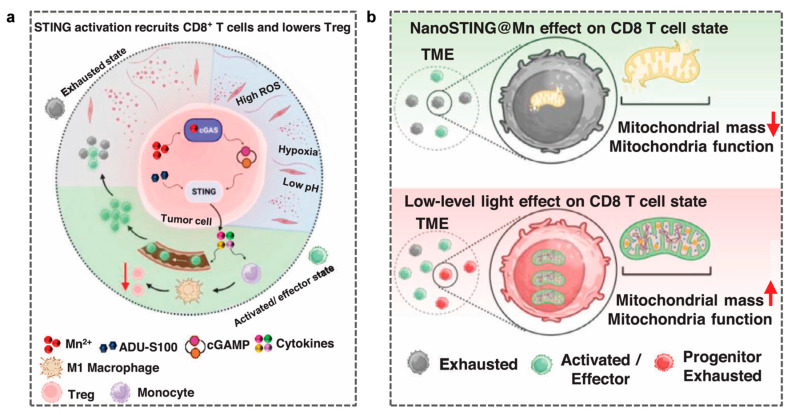
(**a**) Antitumor mechanism of intratumoral injection of nano-STING@Mn [142]. (**b**) The combined use of NanoSTING@Mn and low-level laser (LLL) can eradicate tumors. Reprinted (adapted) with permission from [142]. In the upper part of the figure, the downward arrow indicates a decrease in Mitochondrial mass, and in the lower part, the upward arrow indicates an increase in Mitochondrial mass. Reprinted (adapted) with permission from Ref. [142]. Copyright 2026, copyright Banstola A, Gao S, Zhang Z, et al.

Low-level light (LLL) therapy, a form of photobiomodulation, is an innovative, clinically practical, non-invasive, and drug-free treatment method that directly restores mitochondrial function in tumor-infiltrating CD8^+^ T cells. Unlike traditional drug formulations that rely on systemic distribution (which is often impaired in tumors), LLL directly enhances the mitochondrial respiratory chain activity by activating complexes I, III, and IV of the electron transport chain, thereby converting light energy into bioenergy in the form of ATP within the inner mitochondrial membrane [136,137]. As ATP production is light-driven, it relies less on oxygen and remains effective in ischemic or near-hypoxic tissues, as confirmed by both in vivo [138,139] and in vitro [140,141] experiments (Figure 7a).

Banstola et al. proposed a dual-strategy combining LLL therapy with a nano-sized STING agonist formulation (nanoSTING@Mn) [142], which effectively activates the cGAS-STING pathway, induces a type 1 IFN response, and promotes lymphocyte infiltration (Figure 7b). These monocytes polarize into M1 macrophages, which suppress regulatory T cells. Concurrently, LLL photobiomodulation reprograms mitochondrial metabolism in tumor-infiltrating CD8^+^ T and NK cells, restoring their persistence and potentially leading to the complete eradication of local tumors.

#### 4.2.6. Multimodal Collaborative Therapy

The therapeutic efficacy of Mn-based immunotherapy alone is limited by tumor heterogeneity and diversity as well as the complex immunosuppressive TME. To enhance the efficacy of tumor treatment while minimizing drug dosage and side effects, immunotherapies involving multimodal synergistic therapies have been developed that combine Mn-based nanomaterials with various therapeutic agents. Recently, multimodal synergistic therapies based on Mn-based nanomaterials have overcome the limitations of single-modality therapies in addressing tumor heterogeneity and the immunosuppressive TME by integrating multiple mechanisms, including PTT, PDT, CDT, starvation therapy, and immune activation, thereby achieving a synergistic effect.

**Figure 8:**
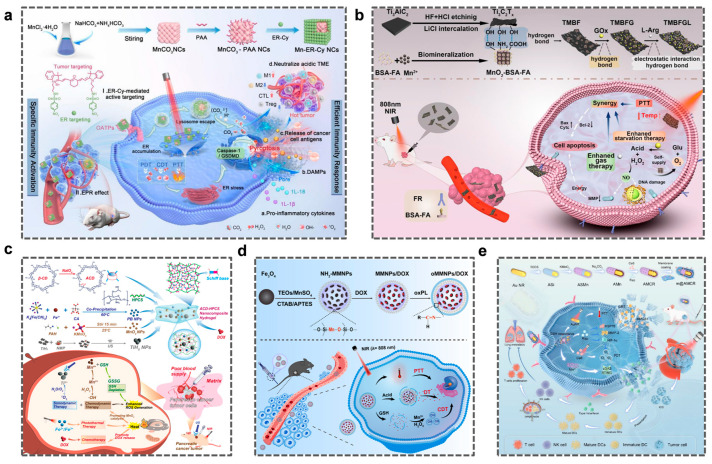
(**a**) Schematic diagram of the preparation of Mn-ER-Cy and its anti-tumor mechanism of multi-modal synergistic therapy through photothermal therapy (PTT)/photodynamic therapy (PDT)/chemodynamic therapy (CDT). Reprinted (adapted) with permission from [143]. Copyright 2025, copyright Wu C, Gao M, Xiao W, et al. (**b**) Schematic design and anti-cancer mechanism of a robust Ti_3_C_2_T_x_ MXene cas-cade catalytic system for tumor therapy. Reprinted (adapted) with permission from [144]. Copyright 2026, Wiley. (**c**) ACD-HPCS injectable hydrogel loaded with PB, TiHx, MnO_2_ nanoparticles and doxorubicin (DOX) for synergistic photothermal therapy (PTT)—sonodynamic therapy (SDT)—chemodynamic therapy (CDT)—chemotherapy (CT) in the treatment of pancreatic cancer. Reprinted (adapted) with permission from [145]. Copyright 2026, American Chemical Society. (**d**) Synthesis and drug release of a pH/glutathione/near-infrared triple-triggered drug carrier. Copyright 2026, American Chemical Society. Reprinted (adapted) with permission from [146]. Copyright 2024, American Chemical Society. (**e**) The mechanism by which m@AMCR induces a strong immune response through photodynamic therapy (PDT)/photothermal therapy (PTT)-mediated immunogenic cell death (ICD) and activation of the stimulator of interferon genes (STING) pathway for tumor treatment. Reprinted (adapted) with permission from [149]. Copyright 2026, American Chemical Society.

Mn-ER-Cy nanocubes integrate PTT/PDT/CDT tri-modal therapy via endoplasmic reticulum targeting (Figure 8a), inducing NLRP3 inflammasome activation and ICD and modulating the TME pH, thereby enabling imaging-guided precision therapy and significantly enhancing tumor immunotherapy efficacy [143]. The multifunctional manganese-based nanozyme developed by Yang et al. uses Ti_3_C_2_Tx MXene nanosheets as carriers and is surface-functionalized with MnO_2_ modified by FA and BSA. An efficient cascade catalytic system is designed: Ti_3_C_2_Tx MXene+MnO_2_-BSA-FA+GOx+LArg (TMBFGL), which has dual responsiveness to acidic pH values and H_2_O_2_. It achieves synergistic anti-tumor effects through photo-thermal, photodynamic and immune mechanisms, providing a safe and effective platform for reversing the immunosuppressive microenvironment and realizing synergistic anti-tumor immunotherapy (Figure 8b) [144]. Even the ACD-HPCS hydrogel integrates Prussian blue NPs as a photothermal agent, titanium hydride NPs (TiHx NP, x ≈ 2–4) as a sonosensitizer, MnO_2_ NP, and DOX (Figure 8c). It combines NIR-induced PTT, ultrasound SDT, and CDT to achieve a four-modal synergistic therapeutic effect combining PTT, SDT, CDT, and CT [145].Meanwhile, the oMMNPs/DOX nanoplatform developed by Li et al. uses magnetic targeting and a triple-responsive mechanism (pH/GSH/NIR) to synchronously release DOX and Mn^2+^ (Figure 8d). It has significant antitumor potential in PTT, CDT, and integrated diagnosis (MRI/fluorescence dual-mode imaging), with a tumor suppression rate 65% higher than that of single-modality therapies [146]. This platform possesses excellent drug release control capabilities, good biodegradability, precise targeted drug delivery, and high photothermal conversion efficiency, offering important research directions and application prospects for the field of cancer therapy. MGF@laN NPs release Ga^3+^ (inducing mitochondrial apoptosis) and Mn^2+^ (activating the cGAS-STING pathway) in a pH-responsive manner, synergistically disrupting the ionic homeostasis of tumor cells and enhancing ICD. Simultaneously, by leveraging the β1-integrin on neutrophil membranes to block the formation of metastatic niches, this study first proposed and achieved a triple synergistic therapy combining apoptosis, immunogenicity, and metastasis inhibition [147]. rGOQD/MnO_2_/GOx/CPP NPs integrate a triple-modality therapy combining chemodynamics (MnO_2_-catalyzed H_2_O_2_ generation of ·OH), starvation (GOx-mediated glucose oxidation depleting ATP), and photothermal effects (rGOQD near-infrared response) to synergistically disrupt tumor antioxidant defenses (GSH depletion) and heat shock protein expression, achieving highly effective treatment of U87 glioblastoma [148]. More notably, m@AMCR NPs, loaded with chlorin e6 and rapamycin (Rap), combine PDT/PTT with cGAS-STING pathway activation (Figure 8e) [149]. YangQi et al. designed a multifunctional manganese (Mn)-based nanozyme, Ti_3_C_2_-MnO_2_-PDA, which responds to the acidic pH and overexpresses H_2_O_2_ at the tumor site and has the ability to regulate the hypoxic and immunosuppressive tumor microenvironment (TME) for synergistic antitumor photothermal/photodynamic/immunotherapy [150]. Addressing the limitation of CDT caused by excessive GSH expression in the TME, Cai et al. developed a hollow MnO_2_ (HMnO_2_) multifunctional bioreactor loaded with cisplatin for synergistic CDT-chemotherapy [151].

The above text explains the specific structure, mechanism of action, and therapeutic effects of the synergy between manganese-based nanomaterials and other modalities. Below, the remarkable data results of the materials are described in detail. For example, Mn-ER-Cy can actively and precisely target the ER organelles of tumor cells, thereby enhancing the local therapeutic effect [143]. Moreover, compared with conventional systems (usually less than 40%), Mn-ER-Cy achieved a significantly higher photothermal conversion efficiency (51.6%) under 808 nm irradiation. Although the preliminary biosafety assessment indicates low acute toxicity, it is crucial to conduct a comprehensive assessment (including studies on chronic toxicity, immunogenicity, metabolism, and excretion) before clinical application. The data obtained during the development of Ti_3_C_2_Tx MXene show that the migration rate of the pure GOx group decreased but remained at 75.58% [144]. After loading GOx onto Ti_3_C_2_Tx MXene, the cell migration rate was 62.07%. Subsequently, loading L-Arg led to a further decrease in cell migration to 50.13%. After irradiation with an 808 nm laser, the cell migration rate further decreased to 28.49%. The experimental results of pancreatic cancer mice treated with TiHx NPs showed the strongest tumor inhibition [145]. The final tumor volume was only 130.22 ± 23.3 mm^3^ (approximately 18.3% of the final volume of the control group), and the tumor weight was 0.10 ± 0.02 g, which was significantly smaller than that of other groups (*p* < 0.001), and there was no difference in the body weight of the mice compared with the control group. The experimenters tested the anti-cancer efficacy of NH_2_-MMNPs and oMMNPs/DOX in a mouse model. The combination therapy group (oMMNPs/DOX + NIR) showed significant inhibition of tumor growth, with a tumor inhibition rate of 84.1% [146]. In addition, in the experiment, the m@AMCR + 638/808 nm light group showed the strongest tumor-inhibiting effect (tumor inhibition rate of 87.39%), which was 1.34 times that of the AMCR + 638/808 nm light group. This result can be attributed to the excellent tumor-accumulating ability of m@AMCR [149], leading to enhanced PDT and PTT efficacy. These specific and distinct data show that manganese-based nanomaterials are a promising new approach for cancer treatment.

These studies confirm that Mn-based multimodal synergistic therapy, through the integrated convergence of catalytic oxygenation, immune activation, metabolic reprogramming, and physical killing, not only overcomes the barrier of tumor heterogeneity, but also provides a tunable nanoscale platform for personalized therapy. Future research should further explore the temporal regulatory mechanisms and clinical translation pathways that are expected to lead to the development of more multimodal synergistic therapies involving immunotherapy to achieve personalized cancer treatment (Table 2).

## 5. Summary and Prospect

As an essential trace element for human survival, Mn exhibits valence state conversion responsiveness, paramagnetism, and biodegradability, demonstrating its significant potential in tumor immunotherapy. Based on the characteristics and functional advantages of Mn-based materials, this review thoroughly explored the four-fold mechanism of action in antitumor immunotherapy: activation of the STING pathway, activation of immune cells, induction of ICDs, and regulation of the TME. Regarding STING pathway activation, Mn^2+^ triggers a burst of IFN-α/β by enhancing the sensitivity of cGAS to dsDNA/mtDNA and increasing the affinity between STING and CDNs, thereby driving DC maturation and CD8^+^ T cell infiltration. In terms of immune cell regulation, Mn-based materials directly activate NK cell cytotoxicity and promote M1 polarization of macrophages, and simultaneously enhance T-cell responses via the TCR-ZAP70 signaling pathway. ICD induction relies on ROS generated by MnO_2_ catalysis to trigger endoplasmic reticulum stress, promoting calreticulin externalization and HMGB1 release. TME regulation focuses on reversing hypoxia and lifting immune suppression by degrading adenosine and inhibiting HIF-1α to reshape “cold tumors.” At the application level, Mn-based monotherapy focuses on nanocarriers, CDT, and STING pathway-dependent immune activation. Multimodal synergistic strategies achieve a synergistic effect by integrating chemotherapy, RT, PDT, SDT, low-level laser (LLL) therapy, and multimodal therapies. For example, Mn-based therapy combined with chemotherapy reverses chemotherapy resistance, RT synergy relies on timing control to match the DNA damage window, and PDT/SDT synergy enhances ROS generation through catalytic oxygen production and cavitation effects, simultaneously activating the STING pathway. Multimodal platforms such as HMnO_2_@TPyP@Bro integrate catalytic oxygenation, gas therapy, and MRI guidance, while MGF@LaN NPs achieve a three-tiered synergy of apoptosis, immunotherapy, and metastasis inhibition.

Although Mn-based materials demonstrate significant advantages in tumor immunotherapy, several challenges remain in their clinical translation, and further optimization of Mn-based nanoplatforms is crucial. First, biocompatibility and toxicity are the major limiting factors. Although many studies have indicated that Mn-based nanomaterials have good biocompatibility and low toxicity, these assessments are largely based on in vitro cellular experiments and lack long-term in vivo safety data. Existing research has primarily focused on in vitro cellular models, while the dynamic distribution of Mn^2+^ in vivo, such as blood–brain barrier permeability, long-term accumulation effects, and mechanisms of neurotoxicity, remains unclear. Although surface modification can reduce nonspecific uptake, there is still controversy regarding the metabolic pathways of Mn-based NPs in different tissues and their size-dependent behavior. The association between tumor STING pathway mutation rates and Mn responsiveness remains unclear, and multi-omics analysis is needed to screen for biomarkers to further guide the adaptation of Mn-based carriers. Manganese-based materials have shown significant commercialization potential in the field of tumor immunotherapy. Their core advantage lies in the efficient activation of the cGAS-STING pathway through the controlled release of Mn^2+^, which significantly enhances the anti-tumor immune response, especially suitable for patients resistant to PD-1/PD-L1 inhibitors. As an integrated diagnosis and treatment platform, manganese-based MRI contrast agents can replace gadolinium agents, reducing costs by 30%, and the raw material cost is less than 1% of that of gold-based materials. However, clinical translation faces three major challenges: the risk of neurotoxicity; the bottleneck of large-scale production; and regulatory complexity. Future development directions include combination regimens for reversing drug resistance, second-generation intelligent responsive materials, and innovative clinical endpoint designs to further improve efficacy and safety. Although breakthroughs are still needed in long-term neurotoxicity control and production process optimization, based on their unique immune activation efficacy and cost advantages, manganese-based materials are expected to become a key component in the combined treatment of solid tumors within 5–8 years. The following are three recommendations for the future development of Mn-based materials. First, the development of time-controlled release systems, such as radiation- or ultrasound-triggered smart drug delivery systems, will further improve the precision and safety of treatment. Second, strategies for mitigating neurotoxicity are key to the clinical translation of Mn-based materials. By optimizing material design and surface modification, and elucidating the balance between Mn^2+^ penetration of the blood–brain barrier and STING activation in microglia, potential neurological side effects can be reduced. Furthermore, in-depth research into the mechanisms of action of Mn-based materials across different tumor types and exploration of their potential for personalized therapy will provide a stronger evidence base for clinical practice. Multimodal synergistic therapy is a major direction for future development. By rationally combining different treatment modalities to fully leverage their respective strengths and achieve synergistic effects, Mn-based immunotherapy will transition from a “tool-based enhancement” approach to a “mechanism-driven paradigm.” This will ultimately drive a paradigm shift in cancer treatment from local control to systemic eradication with the potential to further improve treatment efficacy. Future research will further elucidate the potential of Mn-based materials in tumor immunotherapy, providing cancer patients with safer and more effective treatment options.

## Figures and Tables

**Table 1:** Overview of manganese-based nanomaterials for standalone cancer treatment.

Manganese-Based Nanosystem	Ingredient	Mechanism	Tumor Types	Remarks
MnO_2_@CeO_2_-Vin-Ser	ceriumoxide; vincristine; sericin; MnO_2_	Drug delivery	Lung cancer	It can achieve controlled drug release, but it is also necessary to improve the stability of the carrier and evaluate the in vivo efficacy.
MRPH	RRX-001; Mn-ZIF8; PEG-HA	Drug delivery	Osteosarcoma	It can control the release of drugs and activate the cGAS-STING pathway, but long-term safety assessment is still required to determine the safety threshold.
MS@Yeast	manganese silicate nanoparticles; living yeasts	Drug delivery	Breast cancer	It can achieve drug delivery and trigger an anti-tumor immune response.
CS-metformin@MnO_2_ particles	a metformin-loaded chitosan (CS) inverse opal core; MnO_2_	Drug delivery	Breast cancer	Drug-controlled release and tumor immunotherapy with TME-responsive approach
MnO_2_-ICG@BSA	indocyanine green; bovine serum albumin–manganese dioxide complex	Drug delivery; PTT-PDT	Melanoma	It has anti-cancer properties and low toxicity, but long-term safety testing is still required.
MMP NDs	Mn^2+^; MoO_4_^2−^; PEG^5k^; 293-Dual^TM^ mSTING cells	metalloimmunotherapy	Breast cancer	It can inhibit tumors and initiate immunotherapy.
PTX/MnO_2_/GOx-Lip-HAs Nanoparticles	soya lecithin; paclitaxel; MnO_2_ nanoparticles; glucose oxidase; hyaluronic acid	CDT	Cervical cancer	It enhances CDT by combining with tumor starvation to inhibit tumors, and the safety can be continuously monitored.
MIL-53(Fe)@MnO_2_	MIL-53(Fe)-NH_2_; KMnO_4_	CDT	Breast cancer	It can enhance CDT to inhibit tumors. Subsequently, it is recommended to combine with other treatment strategies for synergistic treatment.
Gen@mSiO_2_@MnO_2_-PEG nanocomplex	MnO_2_; mesoporous silica core; PEG; genistein	CDT	Pancreatic cancer	It enhances the efficacy of combination chemotherapy and CDT in pancreatic cancer.
NP_MCA_	Ce_6_; MnO_2_	CDT	Breast cancer	It enhances the tumor immune response and enables the synergistic immunotherapy of CDT and SDT.
As-MnZnSX NRs	Arsenic; MnZnS_X_	cGAS-STING pathway	Liver cancer	It activates the cGAS-STING pathway and induces tumor immune responses.
CMP Mn-based NPs	a bacterial STING agonist; cyclic di-AMP (CDA); Mn^2+^	cGAS-STING pathway	Acute myeloid leukemia	It can stimulate the immune response and inhibit tumors.
DSMSNs	chalcogen-hybridized organosilica; Mn^2+^	cGAS-STING pathway	Breast cancer	It activates the stimulator of interferon genes (STING) pathway and enhances the tumor immune response.
TPP-MMONs	triphenyl-phosphine; MnO_2_; organosilica nanoparticles	cGAS-STING pathway	Breast cance	It can effectively inhibit tumors and distant metastases.
CMNP	MnO_2_@BSA; DOX; NR	cGAS-STING pathway	Breast cance	It achieves tumor-targeted therapy and coordinates the immune response by activating the cGAS-STING signaling pathway.
MCCS	Mn^2+^; silk sericin (SS); pentapeptide CREKA; aCTLA-4	cGAS-STING pathway	Lung cancer	It can not only be used for precise tumor treatment but also has biocompatibility and safety.
Mn-LDH-Ce_6_	Mn; Ce_6_	cGAS-STING pathway	Breast cance	It effectively reverses immunosuppression and inhibits tumor growth.

**Table 2:** Overview of manganese-based nanomaterials for synergistic cancer therapy.

Manganese-Based Nanosystem	Ingredient	Mechanism	Tumor Types	Remarks
PL/APMP-DOX NPs	porous manganese phosphate (APMP) NPs; DOX; phospholipid (PL)	cGAS-STING; innate immunity	Breast cancer	It releases DOX and Mn^2+^ inside cancer cells to stimulate the cGAS-STING pathway, thereby inhibiting tumors and enhancing immunotherapy.
OXA/Mn@LCSF	oxaliplatin (OXA); liquid crystal gel formation system (LCFS); MnP@Lip nanoparticles	cGAS-STING; platinum-based chemotherapeutics; immunochemotherapy	Breast cancer	It synergistically promotes anti-tumor and immunotherapy by combining manganese-based agents with platinum-based chemotherapy drugs.
C-NAG-R8-PTXL/MnO_2_-lip	NAG/R8-dual-ligand; O^2−^ producing liposome; MnO_2_	Synergistic chemodynamic; chemotherapy	Lung cancer	It synergized with chemotherapeutic drugs to reverse both basal MDR and hypoxia-induced drug resistance.
RMLF	BSA-MnO2 (MnB); Lipo; [Ru (DIP)_2_dppz]^2+^ (RudZ); folate	Radioimmunotherapy; RT	Breast cancer	It effectively inhibits tumors and stimulates immune responses through the synergy of manganese-based substances and RT.
SOCS6@vHMMn-Bi nanoparticles	virus-inspired hollow mesoporous manganese-bismuth bimetallic oxide nanoparticles (vHMMn-Bi); suppressor of cytokine signaling 6 (SOCS6)	hypoxia relief; RT	Esophageal squamous cell carcinoma	It has considerable potential as a dual-mode radiosensitizer, without systemic toxicity and with low immunogenicity, and can enhance the efficacy of radiotherapy.
Mn-ZIF-8 MNs	Mn^2+^; soluble microneedles (MNs); zeolite imidazolate frame-8 (ZIF-8)	RT; cGAS-STING	Melanoma	It is a microneedle patch with X-ray responsiveness, rapid dissolution and controlled release capabilities, and it can enhance the efficacy of radio immunotherapy for cutaneous melanoma.
αPDL1@MnO_2_	high antiprogrammed death ligand 1 (αPDL1); MnO_2_	RT; cGAS-STING; ICD	Colon cancer	It can effectively inhibit the growth of primary and metastatic tumors and enhance the systemic anti-tumor response.
Bio-MnO_2_ NPs	MnxEFG complex; MnO_2_ nanoparticles (NPs)	RT; cGAS-STING	Lung cancer	It reversed the tumor microenvironment (TME) and synergized with RT to induce an anti-tumor immune response in non-small-cell lung cancer.
MNPT	Ce6; BSA@MnO_2_; dopamine (DPA)-modified Fe_3_O_4_ (DPA@Fe_3_O_4_)	PDT; TME; Immunotherapy	Breast cancer	MNPT improved the effect of PDT, enhanced the killing effect on tumors and immunotherapy.
MnO-N/C NPs	ZnO; polyacrylic acid; Mn	PDT; TME	Liver cancer	It can minimize the damage to normal cells while killing cancer cells.
ICG@MnO_2_@Exo-anti-PD-L1 NPs	ICG; MnO_2_ NPs; azide-choline (AECho)	PDT; Immunotherapy; TME	Lung cancer	It can be precisely delivered to the tumor site and reshape the tumor microenvironment.
PMMAA	PDA@Mo_2_C-MnO_2_-Au/Apt-M	PTT/CDT; TME	Breast cancer; Liver cancer	It can precisely target tumors, ensuring preferential accumulation at the tumor site and minimizing off—target effects.
FM@VP	bioreducible polyamidoamine (PAMAM); verteporfin (VP); MnO_2_	PDT; immunomodulatory	Breast cancer	It can be combined with photodynamic therapy/cancer-targeted therapy and also has immunomodulatory ability.
EcN + UCNPs@mSiO_2_-MnO_2_-ZnPc	C@3S; zinc phthalocyanine (ZnPc); *Escherichia coli* Nissle 1917	PDT; CDT; TME	Cervical cancer	It solves the two major bottlenecks in the traditional combination of PDT and CDT, namely limited tissue penetration and insufficient intratumoral H_2_O_2_ generation. However, the biosafety still needs continuous observation.
MnCO_3_ NPs	MnCO_3_ NPs	SDT; mitochondrial regulation	Breast cancer	It is a sonosensitizer with mitochondrial regulatory ability and shows a high tumor inhibition rate during CDT tumor treatment.
Mn-HAP	Mn; HAP nanorods	cGAS-STING; SDT; PDT; TME	Breast cancer	It releases Mn^2+^ in a slightly acidic environment, which activates the cGAS-STING pathway, induces tumor immunity, and improves the therapeutic effect of adaptive immunity.
NanoSTING@Mn	ADU-S100; Mn^2+^	low-level light (LLL); photo-biomodulation; cGAS-STING	lymphoma	It can inhibit tumors, prevent their metastasis, and also establish systemic anti-tumor immunity.
Mn-ER-Cy	MnCO_3_; photosensitizer (ER-Cy); endoplasmic reticulum (ER)	CDT; PTT; PDT; TME	Breast cancer	It inhibits tumor progression and promotes systemic immunity through a three-mode synergy.
MnO_2_-MXene	Ti_3_C_2_Tx MXene+MnO_2_-BSA-FA+GOx+LArg (TMBFGL)	PTT; SDT; PDT	Cervical cancer	It showed significant tumor ablation and anti-migration effects in a series of experiments, with fewer side effects.
ACD-HPCS Hydrogel	aldehyde-modified β-CD (ACD) titanium hydride nanoparticle; DOX; MnO_2_ NP	PTT; SDT; CDT; CT	Pancreatic cancer	It can promote the accumulation of drugs in tumors and achieve the four—mode synergistic therapeutic effect combining PTT, SDT, CDT and CT.
oMMNPs/DOX nanoplatform	DOX; a multifunctional manganese-doped mesoporous magnetic nanodrug carrier (NH_2_-MMNPs)	PTT; CDT; DT	Breast cancer; Cervical cancer	oMMNPs/DOX have the potential utility as magnetically targeted and pH/GSH/NIR triple-triggered drug carriers for synergistic PTT/CDT/DT therapy.
MGF@laN NPs	gallium-based metal–organic framework that encapsulates Mn^2+^ (MGF); liposomes; activated neutrophil membranes (LipaNEM)	Apoptosis; immunity; ICD; metastasis suppression	Breast cancer	This study first proposed an apoptosis-immunity-metastasis inhibition triple cascade for tumor treatment, providing a new option for cancer treatment with metal ion-based biomimetic nanomedicines.
rGOQD/MnO_2_/GOx/CPP nanoparticles	reduced graphene oxide quantum dots (rGOQD); MnO_2_; glucose oxidase (GOx); cell-penetrating peptide (CPP)	ST; CDT; PTT	Glioma	It can significantly inhibit the growth rate of tumors and improve the overall treatment effect.
m@AMCR	MnO_2_; chlorin e6; rapamycin (Rap)	PDT; PTT; cGAS-STING pathway	Breast cancer	It effectively inhibits the growth of primary tumors and lung metastasis in combination with innate immune activation.
Ti_3_C_2_-MnO_2_-PDA	Ti_3_C_2_-Mxene; MnO_2_; KMnO_4_	cGAS-STING; PTT; PDT; immunotherapy	Breast cancer	It has the ability to regulate the hypoxic and immunosuppressive TME and can be used for synergistic photothermal/photodynamic/immunotherapy against tumors.
HMnO_2_@CDDP	HMnO_2_; Na_2_CO_3_; SiO_2_; CDDP	CDT; chemotherapy; TME	Lung cancer	It effectively amplifies the chemotherapy efficacy of CDDP and integrates Mn^2+^-mediated CDT with CDDP-induced chemotherapy.

## Data Availability

No new data were created or analyzed in this study.

## Abbreviations

The following abbreviations are used in this manuscript:

CDT	Chemodynamic therapy
CM	Cell membrane
CTL	Cytotoxic T lymphocyte
DAMP	Damage-associated molecular patterns
DC	Dendritic cell
DOX	Doxorubicin
GSH	Glutathione
ICD	Immunogenic cell death
IFN	Interferon
LLL	Low-light therapy
Mn	Manganese
MRI	Magnetic resonance imaging
NK	Natural Killer
NP	Nanoparticle
PDT	Photodynamic therapy
PTT	Photothermal therapy
PTX	Paclitaxel
ROS	Reactive oxygen species
RT	Radiotherapy
TAM	Tumor-associated macrophage
TCR	T cell receptor
TME	Tumor microenvironment
SDT	Sonodynamic therapy
